# Hyperbaric Oxygen Therapy Improving Penile Calciphylaxis

**DOI:** 10.7759/cureus.9190

**Published:** 2020-07-14

**Authors:** Matthew Lipinski, Nitasa Sahu

**Affiliations:** 1 Internal Medicine, Penn State Hershey Medical Center, Hershey, USA

**Keywords:** penile calciphylaxis, hyperbaric oxygen therapy, end stage renal disease (esrd)

## Abstract

Calciphylaxis is a rare and poorly understood disease that almost exclusively occurs in end-stage renal disease (ESRD). It is characterized by the calcification of medium and small dermal arterioles with resultant gangrenous necrosis. Patients develop exquisitely painful skin ulceration and necrosis, typically in the lower extremities. Treatments are severely limited, and mortality is high, as few treatment options provide a survival benefit. Improvement in a few calciphylaxis cases affecting the extremities or abdomen have been reported using hyperbaric oxygen therapy (HBOT), however, very few have had a favorable response when it affected penile tissue. We present a case of a patient with ESRD on hemodialysis and subtotal parathyroidectomy who had biopsy-proven penile calciphylaxis with refractory pain who ultimately underwent successful HBOT.

## Introduction

Calciphylaxis is a poorly understood disease that almost exclusively occurs in end-stage renal disease (ESRD). It has been reported in 1%-4.5% of patients with ESRD requiring hemodialysis mostly involving the lower extremities, however, penile involvement is quite rare [1]. It is characterized by the calcification of medium and small dermal arterioles with resultant gangrenous necrosis. The pathophysiology is still not well-understood, however, it is considered to be due to phosphate and calcium dysregulation. Patients develop exquisitely painful skin ulceration and necrosis. Treatments are severely limited and include sodium thiosulfate infusions, penectomies, and hyperbaric oxygen therapy (HBOT), however, there are no standard treatment guidelines. Mortality remains high with an estimated six-month survival of 50% [2]. Traditionally, HBOT is used to improve wound healing and has 13 Food and Drug Administration (FDA)-approved indications, including the treatment of air/gas embolism, carbon monoxide poisoning, decompression sickness, thermal burns, and diabetic wounds, with the idea of increasing blood oxygen to improve oxygen delivery to the affected tissue [3]. Using the therapeutic treatment of HBOT for calciphylaxis is not yet defined and as such, we present a case of successful use of HBOT in the treatment for penile calciphylaxis refractory to sodium thiosulfate.

## Case presentation

**Figure 1:**
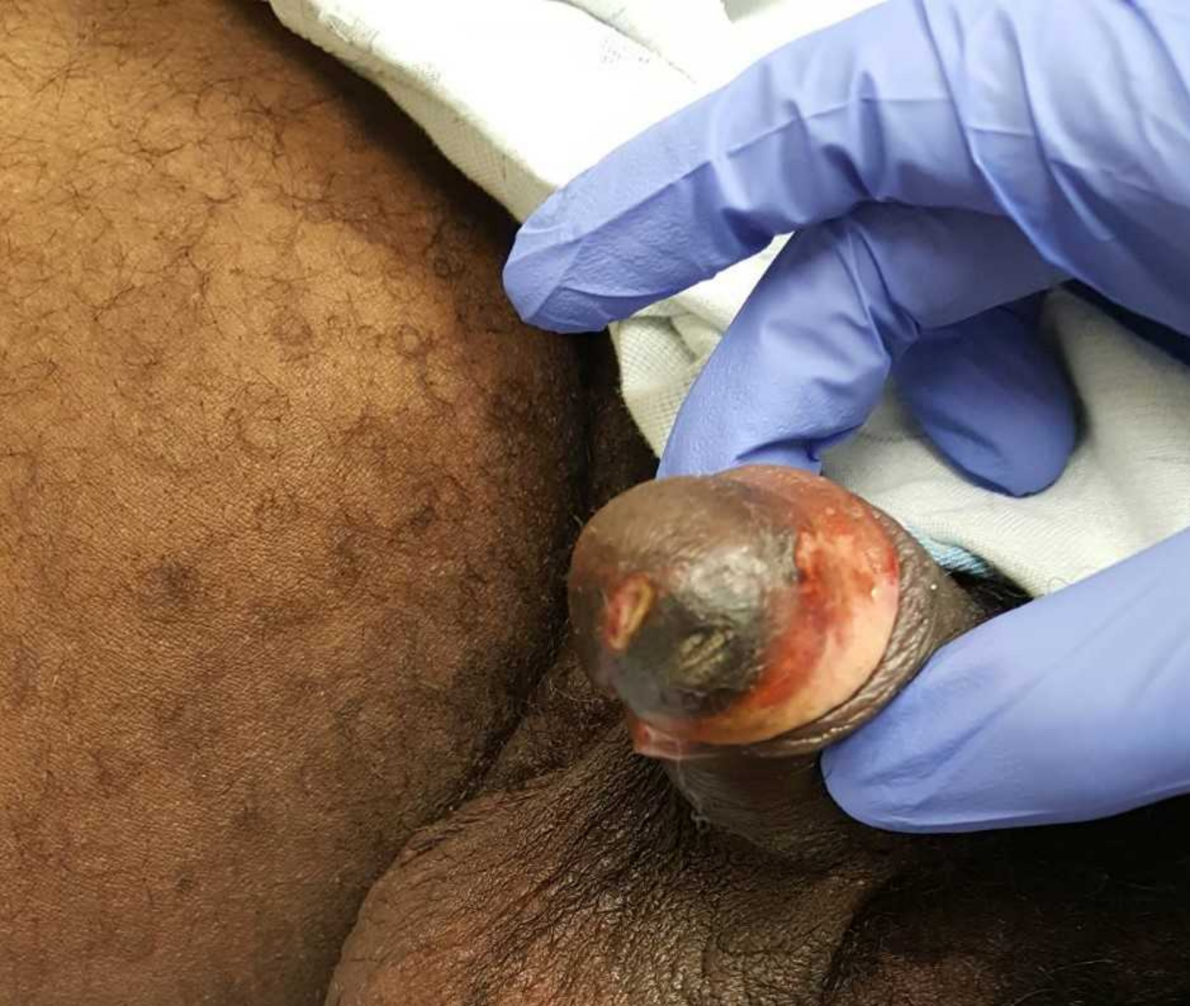
Ulcerated lesions on the glans penis and fibrotic tissue around the corona

A 40-year-old man presented to the hospital with several weeks of penile pain with associated skin lesions. His medical history was notable for ESRD on hemodialysis and parathyroidectomy. He noticed excruciatingly painful ulcerated and necrotic lesions on his penis. He was not sexually active. On physical exam, his penis was symmetrical with normal testes. The glans penis had ulcerated lesions and fibrotic tissue around the corona (Figure 1).

**Figure 2:**
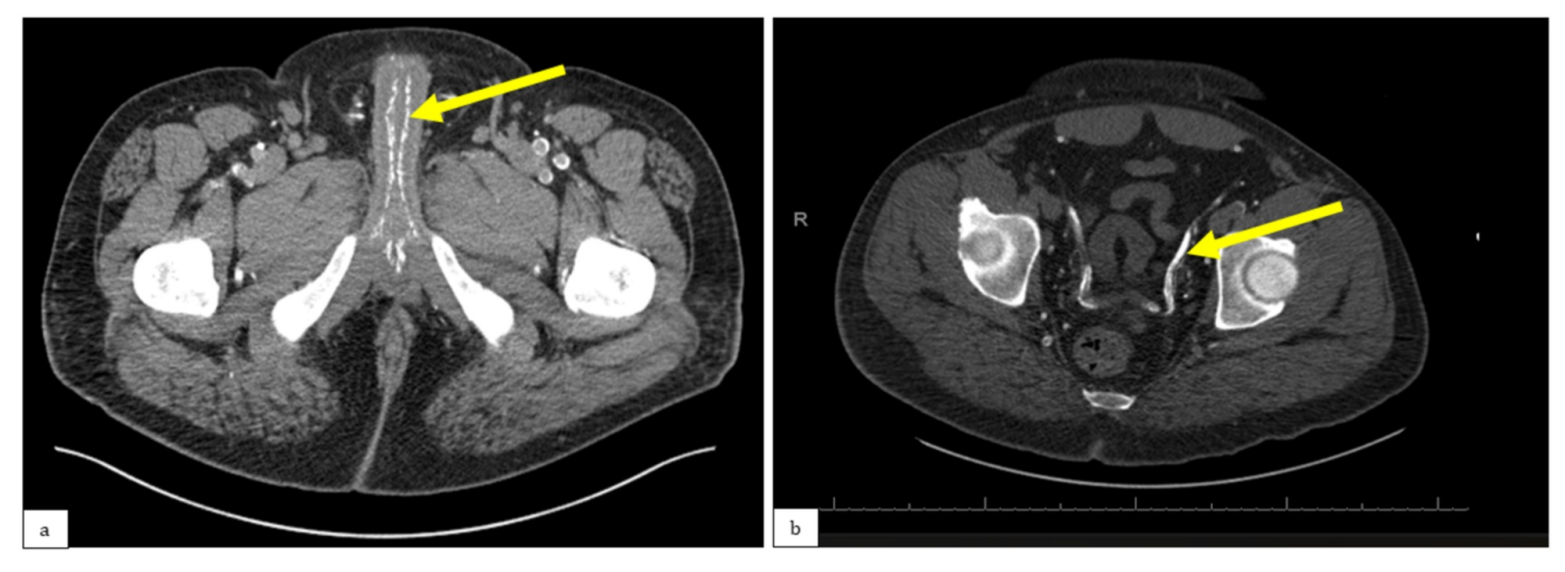
a) Non-contrast CT scan showing extensive calcification of the cavernosal arteries (yellow arrow); b) non-contrast CT scan showing calcification of the vas deferens (yellow arrow) CT: computed tomography

Bloodwork revealed calcium 9.5 mg/dL, phosphorus 8.9 mg/dL, and calcium-phosphorus product 84.6. Parathyroid hormone ranged from 700-1200 pg/mL. A previous outpatient biopsy showed calcification and necrosis in the subdermal vasculature, and computed tomography (CT) of the pelvis showed extensive calcification of cavernosal arteries and vas deferens (Figure 2), consistent with calciphylaxis.

He was admitted for intractable pain, similar to prior hospitalizations. He was continued on hemodialysis and started on sodium thiosulfate with no symptom improvement. His pain was uncontrolled despite topical lidocaine, pregabalin, amitriptyline, and oral opioids. He underwent pudendal nerve blocks, providing temporary relief. Despite local wound care, his symptoms did not improve, and he required continuous high-dose intravenous narcotics. Though not initially covered by insurance, through persistence and appeals, HBOT was finally approved. After multiple HBOT treatments, his pain improved tremendously over the course of three weeks with a slow resolution of his ulcerations.

## Discussion

Significant morbidity and mortality are associated with calciphylaxis, given the poor response to therapy, significant pain, and comorbidities existing in these patients. One review of 34 cases reported 64% overall mortality and mean time to death of 2.5 months [4]. The first steps when a patient is identified as having calciphylaxis, that is, correcting plasma calcium and phosphorous and normalizing parathyroid hormone levels, are necessary due to the implications these imbalances cause. Sodium thiosulfate and parathyroidectomy help regulate the calcium-phosphorus product and have improved pain along with the healing of lesions [1]. Sodium thiosulfate works by chelating calcium to produce calcium thiosulfate that is studied to be more soluble and thus more easily cleared [5]. Surgical debridement has not been shown to improve any outcomes, and penectomy has severe psychological tolls on patients [6]. HBOT has been well-described for treating many thermal or diabetic wounds to reverse tissue hypoxia, however, very few penile calciphylaxis cases have been reported where HBOT was successful. One case reported a patient with bilateral lower extremity calciphylaxis having complete resolution of pain and skin ulcers with 38 sessions of HBOT [7]. An additional case reported an improvement in lower extremity calciphylaxis upon completion of seven weeks of HBOT after failing multiple wound debridements and subtotal parathyroidectomy [8]. In a case series of five patients treated with HBOT, two had complete resolution of necrotic skin ulcers while three had some reduction of necrosis, however, two of those withdrew care due to intractable pain and one required amputation from complications of sepsis [9]. Given our patient’s pronounced improvement in pain and penile lesions, this should be strongly considered in refractory calciphylaxis pain as a standard of practice.

## Conclusions

Calciphylaxis is a challenging condition to treat, given its significant morbidity and mortality. Given the rarity of penile involvement, treatments are typically anecdotal. The few cases that exist with successful HBOT provide some guidance, however, further research is needed to help prove HBOT’s benefit, as it is neither approved by the Centers for Medicare and Medicaid Services nor by the Undersea and Hyperbaric Medical Society. We anticipate that the more case reports such as ours and clinical trials reported showing the benefits of HBOT, the higher chance of it becoming recognized as a successful modality of treatment for this rare condition with high mortality.
